# Use of remifentanil in general anesthesia for neonatal non-cardiac surgery: a case series

**DOI:** 10.1186/s40981-018-0218-6

**Published:** 2018-12-07

**Authors:** Trisana Soontrakom, Mineto Kamata, Norifumi Kuratani

**Affiliations:** 1 Department of Anesthesia, Saitama Children’s Medical Center, Saitama, Japan; 2 Department of Anesthesia, Queen Sirikit National Institute of Child Health, Bangkok, Thailand

**Keywords:** Remifentanil, Pediatric anesthesia, Neonate

## Abstract

**Objective:**

This case series aimed to summarize our experience in usage of remifentanil in neonates undergoing non-cardiac surgery.

**Background:**

Physiology of neonates and infants is different from that of adults. Immaturity of their vital organ systems narrows a safety margin of perioperative management including anesthesia. Remifentanil has favorable characteristics for pediatrics such as short duration of action and rapid elimination. Although remifentanil was introduced into clinical practice since 1996, its application to neonatal anesthesia has not been validated yet.

**Methods:**

This is a 14-month retrospective case series of neonates receiving remifentanil during non-cardiac surgery at a tertiary care pediatric hospital in Japan. Patients’ characteristics, intraoperative data, and complications were retrieved from medical records.

**Results:**

A total of 68 neonates underwent non-cardiac surgery under general anesthesia, of whom 48 received remifentanil. Infusion rate was 0.14 (0.04–0.35) mcg/kg/min (median, range). No intractable adverse events including postoperative apnea were detected.

**Conclusion:**

Remifentanil is generally feasible to neonatal surgical population.

## Introduction

The physiology of neonates and infants are different from that of adults. Immaturity of their vital organ systems narrows the safety margin of perioperative management including anesthesia. Remifentanil has favorable characteristics for anesthesia in neonates such as rapid onset, potent analgesic effect, and rapid elimination. Although remifentanil was introduced into clinical practice in 1996, its application to neonatal anesthesia has not been validated yet. Despite existing literatures showing safety and effectiveness of remifentanil in children, especially in neonates [1–6], it has not gained a broad consensus among pediatric anesthesiologists. Currently, available evidence of the remifentanil usage in neonatal surgical population is mostly from anecdotal case reports.

The purpose of this case series study was to summarize our experiences in order to find out a feasibility of remifentanil use in the neonates and also to find any potential problems.

## Patients and methods

This retrospective single-center case-series study was approved by the Institutional Review Board of Saitama Children’s Medical Center, Saitama, Japan. The need for informed consent was waived. This case series included all neonatal cases receiving remifentanil during non-cardiac surgery over a 14-month period (from January 2017 to February 2018). Patients’ characteristics and intraoperative data including a dosage of remifentanil were obtained from hospital electronic medical and anesthesia records. Patients’ characteristics included age, post conceptual age, body weight, gender, underlying disease (with or without cardiac diseases), diagnosis, and operation. Intraoperative data included anesthetic time, operative time, airway management, ability of tracheal extubation in operating room (OR), dosage of remifentanil, and cardiovascular and respiratory complications. All patients received general anesthesia with neuromuscular blocking agent (rocuronium) and the American Society of Anesthesiologists (ASA) standard monitoring (with or without arterial line). Airway management was endotracheal tube intubation. Data pertaining to a dosage of remifentanil, duration of surgery and anesthesia, hemodynamics, and complications were recorded. Postoperative data included postoperative apnea, re-intubation following immediate postoperative extubation in the operating room, and desaturation.

Average infusion rate of remifentanil was calculated by a total dose per body weight divided by duration of infusion. Data are expressed as mean ± standard deviation (SD), median with range or number (percentage), where appropriate. Data were analyzed using the SPSS program.

## Results

A total of 68 neonates underwent non-cardiac surgery, 46 of whom received remifentanil (Table 1). Patients’ characteristics are shown in Table 2. Twenty-eight out of 46 cases received sevoflurane combined with remifentanil, and the remaining 18 cases received remifentanil with other sedative medications during surgery. While six of them obtained midazolam and another six patients received nitrous oxide for sedation, no sedative drugs were used in the remaining. Infusion rate of remifentanil was titrated according to intraoperative hemodynamics. Hypotension occurred in 11 cases. It was defined as a > 20% decrease of systolic blood pressure compared with the blood pressure before anesthesia. Four patients had preoperative respiratory complications. They were intubated before surgery and were not extubated after surgery. Endotracheal tube was removed in the operating room in 26 cases. Bradycardia occurred in only one case, which recovered spontaneously without medical treatment. For postoperative analgesia, eight cases received acetaminophen and 38 cases received local anesthetic infiltration. There was one case that had postoperative desaturation because of upper airway obstruction by macroglossia and oropharyngeal secretion, which was successfully treated by repositioning and suction. Postoperative apnea was not reported in any cases during the first 24 h after surgery.

**Table 1:** Surgical procedures

Procedures	Number (percentage)
Abdominal surgery	
Explor-lap with small bowel anastomosis	7 (15%)
Colostomy	8 (17%)
Gastrostomy	1 (2%)
Lap Ramstedt’s operation (pyloromyotomy)	1 (2%)
Lap Ladd’s surgery for duodenal atresia	2 (4%)
Lap Diamond surgery	3 (7%)
Laparoscopic surgery	1 (2%)
Release intestinal twisting	1 (2%)
Renal tumor remove	1 (2%)
Anorectal surgery	
Cutback operation	4 (9%)
Thoracic surgery	
Thoracoscopic for esophageal atresia	3 (7%)
Open thoracotomy	1 (2%)
Neurosurgery	
VP shunt	1 (2%)
Craniotomy with remove hematoma	1 (2%)
Repair of myelomeningocele	5 (11%)
Cerebral tumor remove	1 (2%)
Ophthalmology surgery	
Len reconstruction	2 (4%)
Other surgery	
Central catheter insertion	2 (4%)
Percutaneous pulmonary artery angioplasty	1 (2%)

**Table 2:** Neonatal cases given remifentanil infusion during surgery

Age (days)	2 (0–27)
PCA (weeks)	38.9 (31.7–43.7)
BW (kg)	2.91 (1.56–4.10)
Gender (male)	19 (41%)
U/D with cardiac diseases	9 (20%)
ASA	
1	1 (2%)
2	10 (22%)
2e	7 (15%)
3	12 (26%)
3e	11 (24%)
4	0 (0%)
4e	5 (11%)
Duration of surgery (min)	120 ± 78
Duration of anesthesia (min)	189 ± 82
Mean of remifentanil rate (mcg/kg/min)	0.14 (0.036–0.348)
mHR (bpm)	149 ± 15
mMBP (mmHg)	44 ± 8
Time of extubation (min)	15 (6–32)

Data are expressed as mean ± standard deviation, median (range) or number (percentage)

*PCA* post conceptual age, *BW* body weight, *U/D* underlying disease, *ASA PS* American Society of Anesthesiologists Physical Status, *kg* kilograms, *min* minutes, *bpm* beat per minute, *mmHg* millimeter of mercury

## Discussion

According to our case review, remifentanil was used in patients with a variety of backgrounds and diseases, suggesting that it is potentially useful in a broad range of neonatal surgical patients. Remifentanil has a short context sensitive half-time, and its pharmacokinetics is influenced by age including neonates [7]. Therefore, the use of remifentanil in neonate is possibly safer compared to fentanyl which may result in over dosage and require postoperative artificial ventilation. Concerning a safety of remifentanil, numerous studies reported stable hemodynamics during an infusion of remifentanil [4]; however, a bolus dose or a high infusion rate should be avoided in cases having possible dehydration. Generally, a recommended dosage for intubation of remifentanil was about 2 mcg/kg. However, the intubation dose was not included in the average dosage of remifentanil reported in this study. Additionally, remifentanil is useful in premature and full-term infants because it results in rapid emergence and low incidence of postoperative apnea [8]. In the present case series, no cardiovascular complications or respiratory depression was detected postoperatively. Regarding the effectiveness of remifentanil, some studies showed that remifentanil provided better intraoperative anesthesia and analgesia and faster postoperative recovery compared with other opioids [8, 9]. Moreover, remifentanil is useful to control surgical stress response. Therefore, the use of remifentanil is trendy in cardiac surgery [9].

Twenty out of 46 remifentanil-receiving cases were remained intubated postoperatively. There were many reasons why those patients were not extubated in the OR. A decision of an attended anesthesiologist for extubation depended on the times that the surgery started and finished, uneventful intraoperative events, and unsuccessful intraoperative resuscitation. The operations of the patients who remained intubated usually finished at late night and caused critically ill conditions. Most patients required inotropic medications. Another reason was heavy pre-anesthetic sedative administration. Therefore, less remifentanil was intended to use in this group of patients.

Some patients received intravascular acetaminophen or local anesthetic infiltration for postoperative analgesia. Although there are a few evidence of hyperalgesia following the use of a high-dose remifentanil, the dosage administered in neonatal cases can be slightly higher than that in adults [9, 10]. Therefore, future studies regarding hyperalgesia in neonatal populations should be investigated.

Remifentanil is used worldwide especially in the developed country. However, it is not available in many countries specifically in the developing countries because of its high cost (fentanyl 0.1 mg = 202JPY, remifentanil 2 mg = 1080JPY).

The limitation of this study is a retrospective study design. The case series study lacks comparison groups; therefore, collected data may be biased and incomplete. Despite that, this study is useful to generate an area for further studies including an appropriate dose range of remifentanil in neonates.

## Conclusion

In our experiences, remifentanil is generally feasible to the neonatal surgical population. Remifentanil might be beneficial to the patients who had been anticipated to extubate immediately after surgery. No intractable adverse events due to remifentanil were found in our retrospective chart review. The prospective formal clinical research would be warranted to establish the rational use of remifentanil in the neonatal surgical population.
